# Multi-strain bacterial combination mitigates pelvic irradiation-induced gut damage by preserving gut integrity, inhibiting inflammation and apoptosis

**DOI:** 10.1038/s41598-026-47773-3

**Published:** 2026-04-15

**Authors:** Babu Santhi Venkidesh, Meghana Acharya, Rekha K. Narasimhamurthy, Thokur S. Murali, Bola Sadashiva Satish Rao, Kamalesh Dattaram Mumbrekar

**Affiliations:** 1 Department of Radiation Biology & Toxicology, Manipal School of Life Sciences, Manipal Academy of Higher Education, Manipal, India; 2 Manipal School of Life Sciences, Manipal Academy of Higher Education, Manipal, India; 3 Central Research Laboratory, Nitte (Deemed to Be University), KS Hegde Medical Academy (KSHEMA), Mangalore, India; 4 Department of Public Health Genomics, Manipal School of Life Sciences, Manipal Academy of Higher Education, Manipal, India

**Keywords:** Radiation-induced gut toxicity, Radioprotection, Gut microbiota modulation, Multi-strain probiotics, Gastrointestinal fibrosis, Anti-inflammatory mechanisms, Quality of life improvement, Microbiome-based therapies, Diseases, Gastroenterology, Microbiology

## Abstract

Pelvic irradiation effectively treats pelvic malignancies, but its side effects can be challenging, causing intestinal damage. Alterations in the gut environment can disrupt the microbiota balance, affecting key microbial communities essential for maintaining gut health. Because the gut microbiota helps maintain gut health, bacterial supplementation may reduce radiation-induced gut toxicity. This study explores the mechanism by which a multi-strain bacterial combination comprising *Lactobacillus, Bifidobacterium*, and *Streptococcus* mitigates pelvic irradiation-induced gut toxicity. Male Sprague–Dawley rats were orally administered a multi-strain bacterial combination throughout the study period or after radiation exposure. Changes in intestinal morphology, integrity, fibrosis, inflammation, and apoptosis were assessed. The prophylactic-therapeutic administration of the bacterial combination effectively preserved villus height, crypt depth, goblet cell count, and overall gut barrier integrity. Furthermore, prophylactic treatment significantly reduced radiation-induced fibrosis, inflammation, and the expression of apoptotic markers in both the jejunum and colon. In contrast, therapeutic bacterial combination treatment was less effective, suggesting that preventive administration is more beneficial in mitigating radiation-induced gastrointestinal damage. Thus, this study underscores the efficacy of pre-radiation bacterial supplementation in protecting the gut from radiation injury, with potential implications for improved patient quality of life.

**Supplementary Information:**

The online version contains supplementary material available at 10.1038/s41598-026-47773-3.

## Introduction

Radiotherapy is a well-established strategy for oncologic management. Approximately 50–60% of cancer patients receive radiotherapy as part of their cancer treatment^1^. Pelvic radiotherapy is commonly used for treating pelvic malignancies, such as urological, gynaecological, and gastrointestinal (GI) cancers and adverse side effects are reported frequently despite continual technological advancements in radiotherapy techniques^2,3^. Following pelvic radiotherapy, damage can occur to the healthy tissues surrounding the tumor, causing acute side effects in 90% of the patients and chronic side effects in 5–10% of the patients, reducing their quality of life^4^. While symptoms of acute radiotoxicity, such as nausea, stomach discomfort, diarrhea, and exhaustion, can appear during the treatment or up to 90 days, one-fifth of individuals receiving pelvic radiotherapy develop chronic radiation enteropathy^5^. Studies also suggest that chronic radiotoxicity symptoms, such as malabsorption, intestinal transit alteration, and dysmotility, could lead to intestinal blockage, fistula formation, intestinal perforation, or pelvic organ dysfunction, occurring even after a latency period of 90 days to several years^6,7^.

The gut microbiota plays vital roles in maintaining the host’s well-being, including nutrient metabolism, digestion, maintenance of the gut environment, detoxification, and modulation of the intestinal immune system. This is achieved by producing small molecules like short-chain fatty acids, tryptophan, and secondary bile acids^8^. Earlier studies have also reported a disturbance in the gut microbial community during and after pelvic irradiation^9,10^. This suggests a possible link between imbalances in the gut microbiota and the adverse effects of radiation.

Various technical and biological approaches have been developed to mitigate radiation-induced toxicity in the GI tract, including prophylactic surgical techniques, optimized planning and delivery techniques, endoscopic therapy, topical therapy (e.g., sucralfate enemas; betamethasone enemas and other agents like mesalazine [5-aminosalicylic acid]) also help reduce inflammation and mucosal damage, and the use of anti-inflammatory agents, antioxidants, and hormones^11–14^; however, the feasibility of these alternatives is constrained by financial limitations, time constraints, and technological availability. Probiotics can be a safe and effective option to maintain healthy gut microbiota and boost gut health. Furthermore, when exposed to radiation, the intestine’s protective ability against invasive pathogens is impaired; thus, bacterial supplementation can help by upregulating the innate immune response in the gut as a part of the protective mechanism against invasive pathogens^15,16^.

Earlier studies^17^ have demonstrated that bacterial supplementation can attenuate gastrointestinal (GI) damage following whole-body radiation exposure in mice. However, these investigations were limited to short-term interventions. In contrast, the current study explores a translational model of pelvic irradiation and evaluates the efficacy of a multi-strain bacterial combination comprising *Lactobacillus, Bifidobacterium,* and *Streptococcus.* Importantly, both prophylactic (pre-radiation) and therapeutic (post-radiation) intervention strategies were assessed, particularly through the restoration of gut integrity and modulation of IL-6 mediated inflammatory and apoptotic signalling pathways, which remain inadequately characterised. Our findings address this gap by studying mechanistic insights into this bacterial combination.

## Results

### Changes in both body weight and fecal consistency

**Fig. 1:**
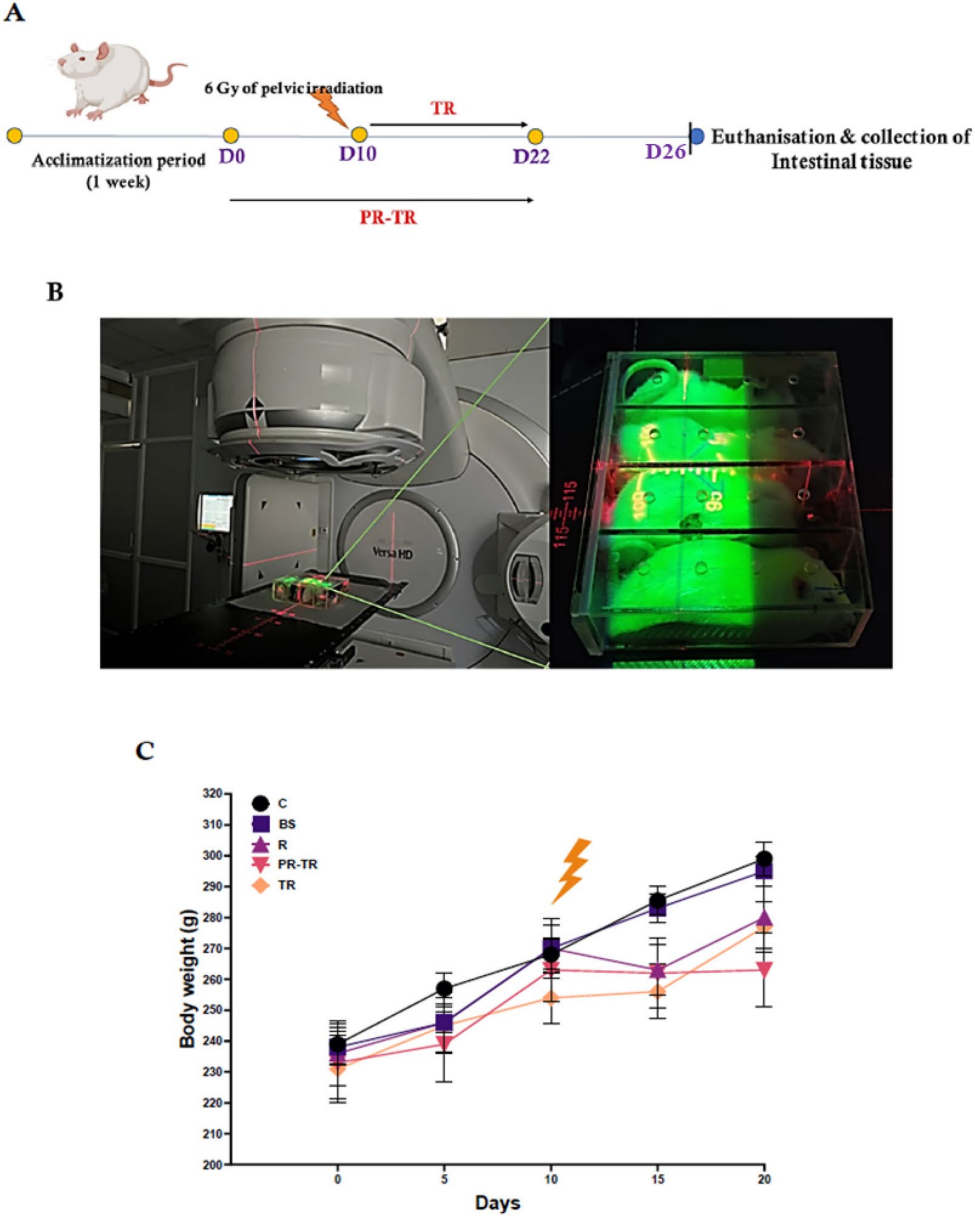
Experimental timeline, irradiation setup and body weight of rats. (**A**) Experimental time line for the current study—Prophylactic-Therapeutic (PR-TR) group was subjected to bacterial supplementation throughout the experiment as well as pelvic irradiation of 6 Gy on the 10^th^ day; and the Therapeutic (TR) group underwent pelvic irradiation on the 10^th^ day followed by bacterial supplementation, (**B**) Photo of the radiation exposure setup, (**C**) Changes in body weight in different groups over the study period. Data are presented as mean ± SEM (n = 6).

No significant changes in body weight were observed across the groups (Fig. 1C). Watery stool consistency was observed in a few animals from the radiation-exposed groups (R, PR-TR, and TR) between days 12 and 16 post-irradiation.

### Ameliorative effects of multi-strain bacterial supplementation on gut morphology

**Fig. 2:**
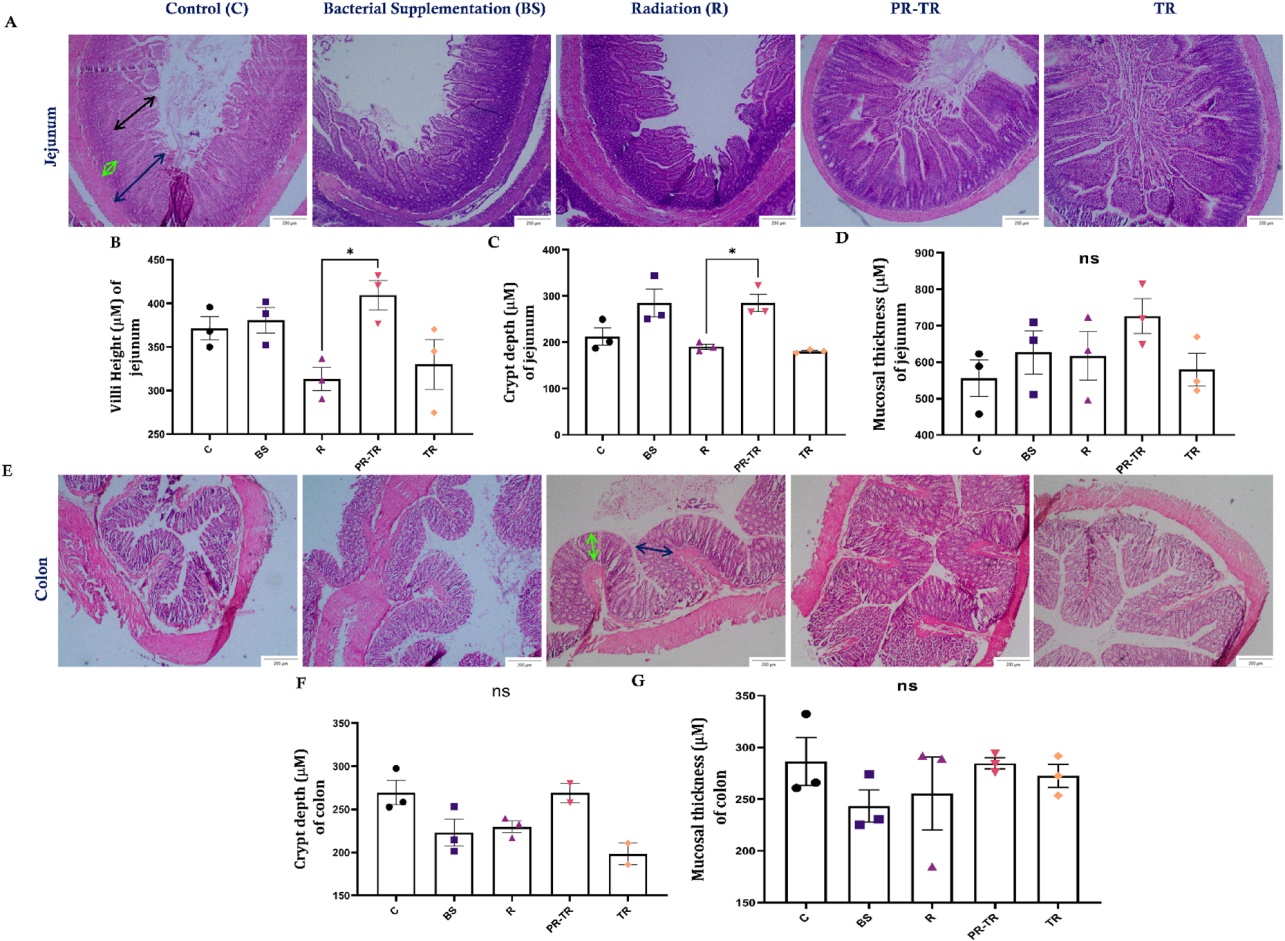
Gut morphological analysis after irradiation and bacterial supplementation treatment. (**A**) Representative images of H&E-stained jejunum sections, (**B**) Villi height of jejunum (**C**), Crypt depth of jejunum, (**D**) Mucosal thickness of jejunum, (**E**) Representative images of H&E-stained colon sections, (**F**) Crypt depth of colon, (**G**) Mucosal thickness of colon, Black arrow represents the villi height, green arrow represents the crypt depth, blue arrow represents the mucosal thickness. Data are presented as mean ± SEM (n = 3). P value * < 0.05, ns- not significant. Scale bar indicates 200 µm (100X). Data are presented as mean ± SEM (n = 3). P value * < 0.05, ns- not significant.

We measured villus height in the jejunum and crypt depth and mucosal thickness in both the jejunum and the colon. Results indicated that the PR-TR treatment led to the preservation (p < 0.05) in villus height (Fig. 2B). Conversely, TR treatment did not result in any notable changes. The crypt depth in the jejunum was maintained (p < 0.05) when BS was given throughout the experiment; no changes were observed in the colon (Fig. 2C). Importantly, in both regions, TR treatment did not exhibit characteristic morphological changes after pelvic irradiation. Further, pelvic irradiation did not affect total mucosal thickness in the jejunum and colon (Fig. 2D). Overall, our results suggest that BS, when given throughout the study, helps protect intestinal morphology, especially the bacterial combination.

#### Intestinal integrity

**Fig. 3:**
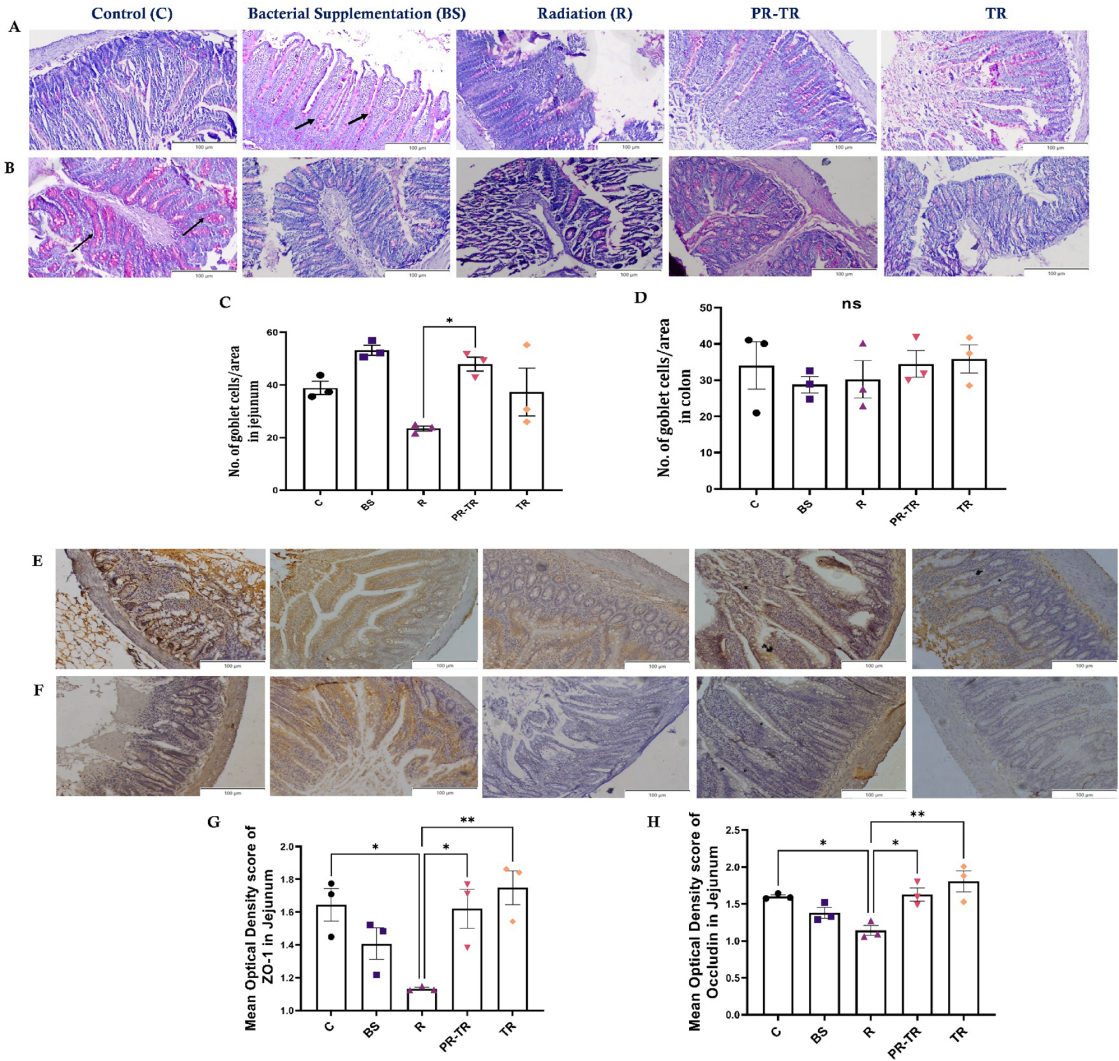
Intestinal integrity of gut after irradiation and bacterial supplementation treatment. (**A**) Representative images for PAS-stained jejunum, (**B**) Representaive image of PAS stained colon, (**C**) Number of goblet cells in jejunum, (**D**) Number of goblet cells in colon, (**E**) Representative images for IHC of occludin in jejunum, (**F**) Representative images for IHC of ZO-1 in jejunum, (**G**) Mean OD score of occludin in jejunum ZO-1 in jejunum, (**H**) Mean OD score of occludin in jejunum. Black arrows represent goblet cells. Scale bar indicates 100 µm (200X). Data are presented as the ± SEM (n = 3). P value * < 0.05, ** < 0.01, ns- not significant.

The effectiveness of bacterial supplementation in mitigating radiation-induced disruption of mucosal integrity was initially evaluated by assessing goblet cell numbers. A substantial rise (p < 0.05) in goblet cell count was observed in the PR-TR group (Fig. 3C). However, radiation treatment did not cause any changes to the colon integrity. Interestingly, in both regions, the TR group showed no characteristic increase in integrity after pelvic irradiation. This suggests that BS has a role in retaining intestinal integrity.

Given the prominence of radiation-induced damage in the jejunum, we assessed intestinal barrier permeability in this region by analyzing tight junction protein expression. The mean optical density analysis indicated that pelvic irradiation reduced (p < 0.01) Occludin and ZO-1 expression in the radiation-alone group compared to the control group (Fig. 3G & H). Both the PR-TR and TR treatment groups displayed significant protection of Occludin and ZO-1 expression compared to the radiation-alone group (p < 0.05 and p < 0.01, respectively). These findings suggest that bacterial supplementation helps maintain intestinal structural integrity and may alleviate radiation-induced barrier disruption.

#### Intestinal fibrosis

**Fig. 4:**
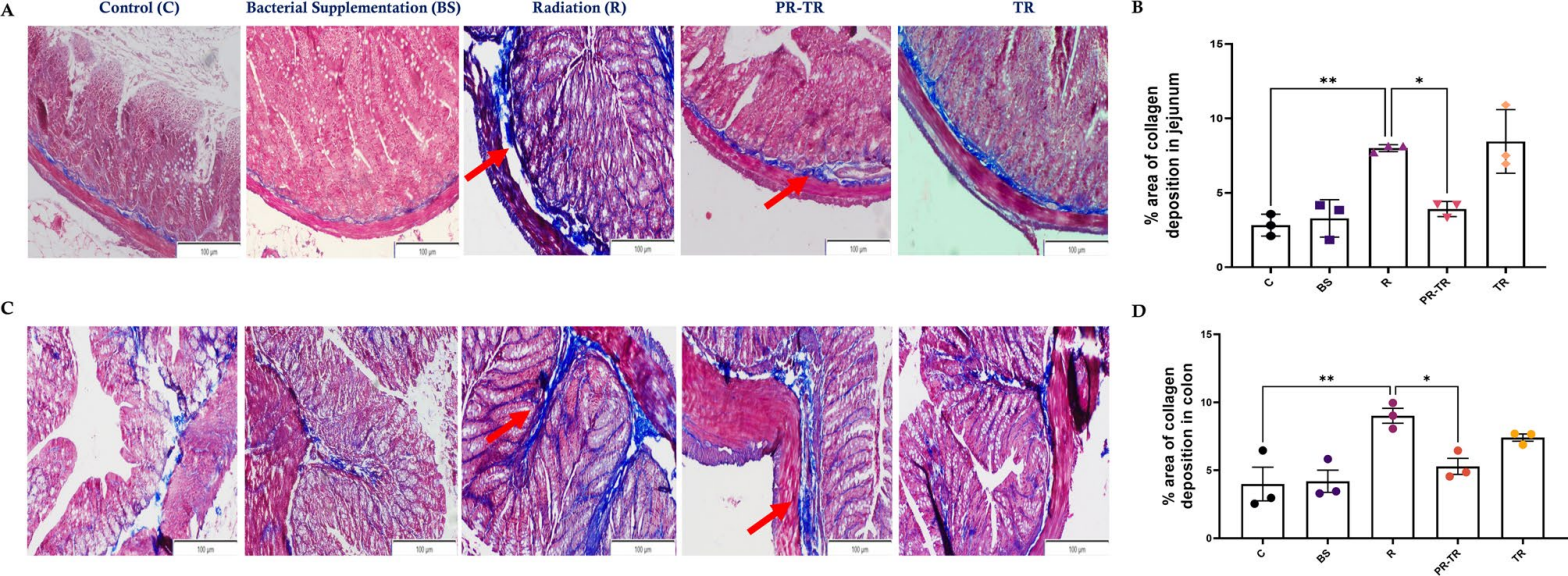
Intestinal fibrosis after irradiation and bacterial supplementation treatment. (**A**) Representative jejunum sections with Masson Trichrome staining, (**B**) Collagen deposition in jejunum, (**C**) Representative colon sections with Masson trichome staining, (**D**) Collagen deposition in colon, red arrows indicate collagen deposition. Scale bar indicates 100 µm (200X). Data are presented as mean ± SEM (n = 3). P value * < 0.05, ** < 0.01, ns- not significant.

Elevated collagen accumulation in the mucosa and submucosa layers of jejunum and colon tissues was observed in radiation-exposed rats (p < 0.01). Intriguingly, the collagen buildup in the PR-TR group of jejunum was reduced (p < 0.05) in comparison to radiation alone (Fig. 4B&D). However, in the TR group, no notable alterations in collagen deposition were observed in either jejunum or colon sections. These findings highlight that 6 Gy of pelvic radiation triggers intestinal fibrosis, and bacterial supplementation throughout radiation treatment can potentially mitigate this fibrotic condition.

### Multi-strain bacterial composition downregulates the genes associated with gut inflammation and apoptosis

**Fig. 5:**
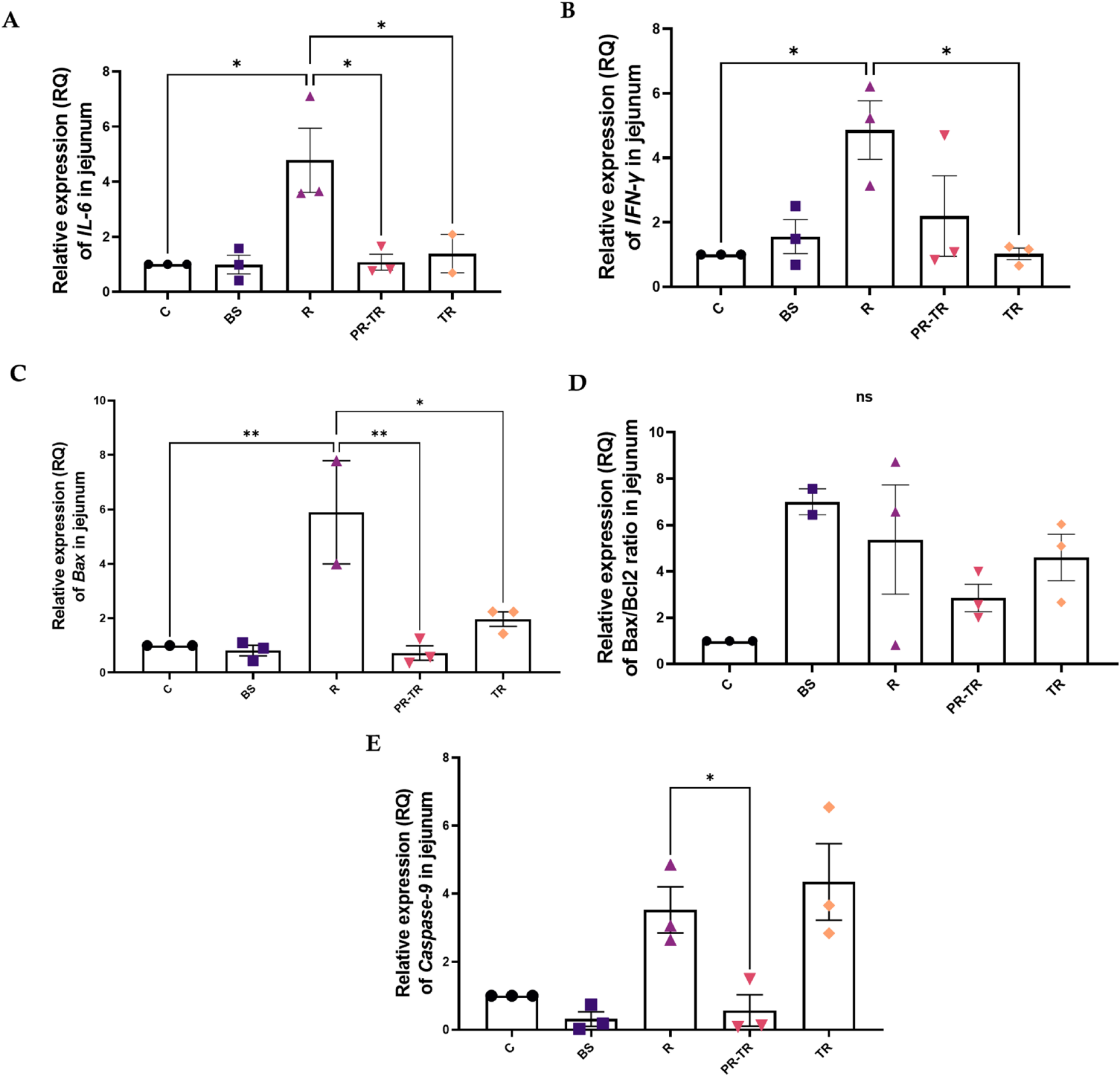
Gene expression for apoptosis pathway genes in jejunum after irradiation and bacterial supplementation treatment. (**A**) *Il-6*, (**B**) *Ifn- γ*, (**C**) *Bax*, (**D**) *Bax/Bcl2* ratio and (**E**) *Caspase-9* in jejunum. Data are indicated as the mean ± SEM (n = 3). P value * < 0.05, ** < 0.01.

To further investigate radiation-induced GI disorders, including gut inflammation and apoptosis, we analysed mRNA expression in jejunum tissues for *Il-6, Ifn-γ, Bax, Bcl-2,* and *Caspase-9.* Reduction (p < 0.01) in *Il-6* expression was observed in the PR-TR group compared to the radiation-alone group (Fig. 5A). Similarly, the TR group also showed a notable decrease in *Il-6* levels (p < 0.05), indicating the anti-inflammatory potential of bacterial supplementation. In parallel, *Ifn-γ* expression was significantly reduced (p < 0.05) in the TR group compared to the radiation-alone group (Fig. 5B), further supporting the immunomodulatory effects of the supplementation. These findings suggest that bacterial supplementation can effectively attenuate radiation-induced intestinal inflammation. Furthermore, Bax, an apoptosis marker, was upregulated in irradiated animals (p < 0.01). In contrast, bacterial supplementation significantly reduced its expression in both the PR-TR (p < 0.01) and TR (p < 0.05) groups (Fig. 5C). However, no significant difference in the *Bax/Bcl-2* ratio was noted among the groups (Fig. 5D). Furthermore, *Caspase-9* expression was significantly attenuated in the PR-TR group (p < 0.05) compared to the radiation-alone group (Fig. 5E), suggesting a protective role of bacterial supplementation against radiation-induced apoptosis.

**Fig. 6:**
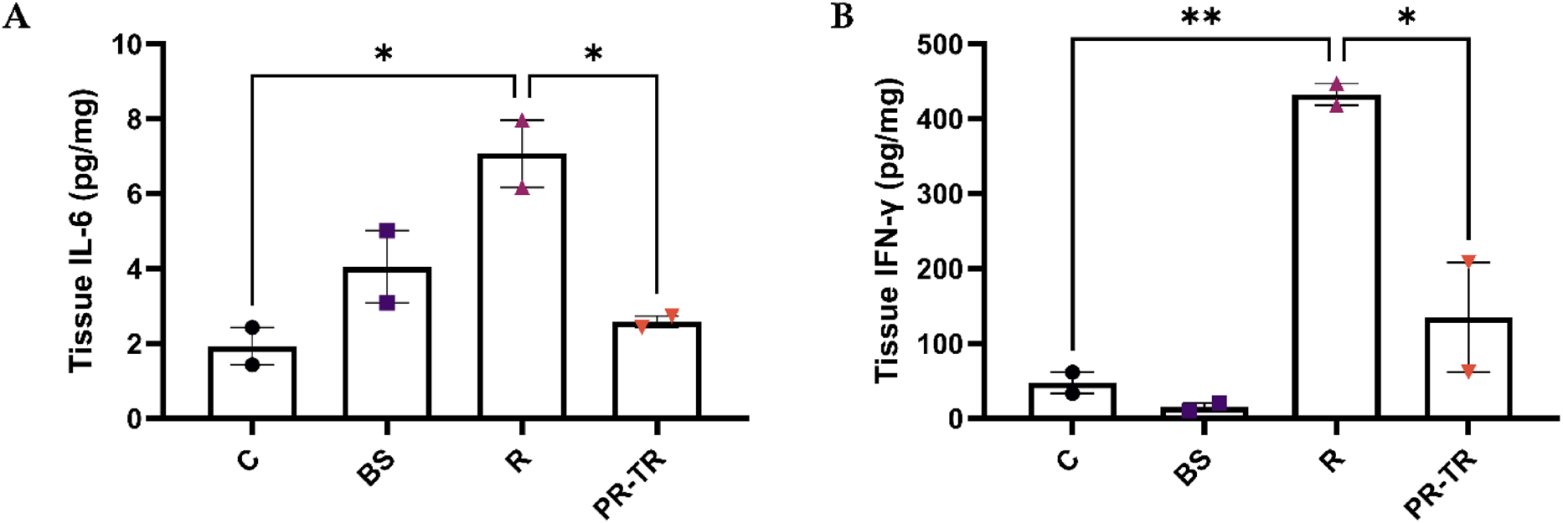
Inflammatory markers in jejunum tissue after irradiation and bacterial supplementation treatment (**A**) IL-6 and (**B**) IFN-γ. Data are indicated as the mean ± SEM (n = 2). P value * < 0.05, ** < 0.01.

To further validate the involvement of inflammatory pathways, IL-6 and IFN-γ protein levels were quantified by ELISA in jejunum tissue lysates, except for the TR group, which did not exhibit significant histopathological alterations. The results showed a significant increase in IL-6 and IFN-γ protein level in the irradiated group compared to the control (p < 0.05 and p < 0.01, respectively). Notably, bacterial supplementation significantly reduced IL-6 and IFN-γ levels in the PR-TR group (p < 0.05) (Fig. 6A & B), reinforcing its role in mitigating radiation-induced intestinal inflammation.

## Discussion

The gut is a highly radiosensitive organ that is vulnerable to the harmful effects of radiation exposure during radiotherapy. It has been reported that radiation can have both short-term and long-term impacts on the GI system^18^. Thus, the present study investigates the underlying mechanisms by which the selected multi-strain bacterial combination mitigates pelvic irradiation–induced gastrointestinal toxicity, with a focus on therapeutic and prophylactic- therapeutic treatment scenarios after 20 days of radiation exposure. The selected bacterial combination comprises functionally significant genera that are prominent members of a healthy gut microbiota. *Lactobacillus* species, known for their ability to metabolise carbohydrates into lactic acid, are major constituents of the lactic acid bacteria group. Their role in aiding the digestion of dietary substrates and providing colonisation resistance against pathogens underscores their symbiotic benefit to the host. Similarly, *Bifidobacterium* represents a dominant genus with a well-established role in early-life microbiota development and maintenance of intestinal health. Numerous studies have demonstrated that a combination of *Lactobacillus*, *Bifidobacterium,* and *Streptococcus* supplementation confers protective effects against intestinal infections and promotes the production of health-promoting metabolites, highlighting their relevance in mitigating gut dysbiosis^19–21^. While the current study demonstrates probiotic efficacy against radiation-induced GI toxicity and inflammation, comprehensive 16S rRNA gene sequencing and metabolomic profiling in our previous study^22^ showed that pelvic irradiation significantly alters gut microbial diversity (Simpson index), while bacterial supplementation partially restores it. The combination treatment helps maintain a stabilised, elevated microbial diversity. Further, the combination group showed pathway enrichment similar to that of the control group, suggesting recovery from radiation-induced metabolic impairment through bacterial supplementation.

Our observations related to radiation-induced changes in intestinal morphology were supported by Mercantepe et al.^23^, who also observed significant villus and crypt loss when exposed to 6 Gy^23^. Bacterial supplementation helped retain villus height in both treatment scenarios. A higher villus height increases the proportion of enterocytes, thereby increasing surface area and promoting beneficial effects, such as enhanced nutrient transport and increased absorptive area^24^. However, in our study, crypt depth analysis in the jejunum region retained its morphology in the PR-TR treatment group compared with the radiation-alone group; no changes in colon morphology were observed. Our findings strongly suggest that BS plays a crucial role in protecting against radiation-induced gut structural alterations following PR-TR treatment. No significant differences in villus height, crypt depth, or mucosal thickness were observed between the control and radiation-only groups, suggesting recovery from radiation-induced damage over time, given the 22-day interval between exposure and sacrifice.

Additionally, PR-TR treatment appears to have a beneficial effect in preserving intestinal morphology. As the gut microbiota play a significant role in maintaining gut health, alterations in the gut environment due to pelvic radiation lead to dysbiosis, which was supported by our study, where we showed that, the pelvic radiation altered the key genera such as *Alloprevotella, Prevotellaceae, Bacteroides, Quinella,* and *Eubacterium* but restored following bacterial supplementation^22^.

Intestinal integrity is maintained by mucus produced by goblet cells in response to microbial metabolites, such as SCFAs, which provide an essential physical and chemical barrier^25^. Akpolat and co-workers observed an initial increase in goblet cells in rats sacrificed 6 h after irradiation, suggesting an immediate protective response. However, in rats sacrificed 4 days post-irradiation, goblet cell numbers declined, suggesting that the protective response may not be sustained over time^26^. This implies that during this period, the death rate of goblet cells exceeds their rate of renewal, leading to goblet cell depletion. Similarly, our study showed that the jejunum goblet cell count was significantly decreased in the radiation group. Studies have also shown that goblet cell number is maintained with bacterial supplementation. For instance, in a recent study, probiotic supplements containing lactic acid bacteria and yeasts were shown to increase the number of goblet cells. Additionally, bacterial supplementation increased goblet cell counts in the large intestine and influenced their differentiation by enhancing the synthesis of acid mucin in the intestinal tract of piglets^27^. Interestingly, probiotic supplementation increased the number of goblet cells in the jejunum without a corresponding significant increase in mucosal thickness. This likely reflects early epithelial differentiation and mucosal functional recovery, whereas restoration of overall mucosal architecture requires longer structural remodelling of villi and crypts following radiation injury. Thus, bacterial supplementation helps maintain goblet cell numbers, thereby preserving the integrity of intestinal tissues. The selective barrier function of the intestinal epithelium is maintained by claudins, Occludin, and ZO-1, which collectively form the TJ barrier. Numerous strains of probiotics, such as *Lacticaseibacillus rhamnosus, Lactobacillus plantarum, and Escherichia coli Nissle* 1917, have been demonstrated to protect the integrity of the intestinal barrier. They achieve this by restoring the levels of tight junction proteins such as claudin-1, Occludin, ZO-1, and ZO-2^28,29^, encouraging the production of mucin^30,31^, reducing inflammation, and supporting the healing of the intestinal lining. In this study, pelvic irradiation reduced Occludin and ZO-1 expression, while bacterial supplementation in the PR-TR and TR groups restored their levels. This suggests that bacterial supplements protect intestinal integrity and help mitigate leaky gut after radiation.

Collagen buildup is invariably preceded by perivascular edema, loss of intestinal structure, and replacement by thick extracellular matrix deposition and severe luminal inflammatory cell infiltration, which are hallmarks of fibrotic areas^32^. Our study showed that radiation induces intestinal fibrosis through excessive collagen deposition. Similarly, Kim et al.^33^ have shown that abdominal radiation induces fibrosis in the intestine^33^. The bacterial supplementation-treated group showed less collagen deposition than the radiation group in both the jejunum and colon. Bacterial supplementation modulates the immune system and maintains a balanced state, thereby reducing fibrosis^34^. Chronic inflammatory responses from radiation-induced tissue damage eventually lead to fibrosis^35^. The significant role of bacterial supplementation in controlling inflammation has been extensively studied in various in vitro and ex vivo models^36^. Bacterial strains such as *Bifidobacterium infantis, Lactobacillus GG, L. acidophilus, L. casei rhamnosus, L. reuteri, and L. sporogenes* have been shown to modulate cytokine production and exert immunomodulatory effects^37^. Therefore, bacterial supplementation might have a potential positive impact in indirectly mitigating intestinal fibrosis.

Our study demonstrated that the group that combined BS with radiation reduced gene expression levels of both IL-6 and IFN-γ, suggesting a potential alleviating effect of BS on radiation-induced intestinal inflammation. Increasing evidence indicates that cytokines, particularly pro-inflammatory ones, play crucial roles in regulating intestinal epithelial homeostasis during inflammation^38^. Pro-inflammatory markers such as IFN-γ have been shown to negatively affect intestinal barrier function and impair the self-rejuvenation of the intestinal epithelium, thereby exacerbating intestinal inflammation^39,40^. Similarly, cytokines such as IL-6 and IL-22 have been implicated in promoting intestinal toxicity by activating the Signal Transducer and Activator of Transcription-3 (STAT3) pathway^41,42^. Further, supplementation with *Bifidobacterium bifidum* reduced IL-6 levels in colon, demonstrating its anti-inflammatory effects^43^. This suggests that BS may play a protective role against radiation-induced intestinal inflammation by modulating key inflammatory pathways. Interestingly, a discrepancy was observed between IL-6 mRNA expression and protein levels. Such differences may arise due to temporal variation between transcription and protein secretion, post-transcriptional regulation, or transient immune activation induced by probiotic components. Since the elevation was not significant and considerable inter-animal variability was observed, this increase likely reflects a short-term adaptive immune response rather than persistent inflammation.

Radiation induces intestinal damage by initiating apoptosis, leading to cell death in affected tissues. Additionally, several studies suggest that bacterial species, such as Lactobacillus, influence the activation of apoptotic and pro-apoptotic signalling pathways^44^. When *Bax* is overproduced, it forms homodimers that promote apoptosis, whereas excess *Bcl-2* results in the predominance of *Bcl-2* homodimers, protecting cells from death^45^. Our findings demonstrated that BS significantly reduced radiation-induced Bax expression. Although we did not observe any difference, the Bax/Bcl-2 ratio acts as a cell death switch, influencing cellular resistance to apoptotic stimuli. A higher *Bax/Bcl-2* ratio decreases resistance, promoting increased cell death and reducing tumour incidence, highlighting the importance of this ratio in determining cell fate in response to apoptotic signals^45^. Further, caspases constitute a group of endopeptidases crucial for the deliberate initiation, execution, and control of apoptosis. Notably, the current study showed that the PR-TR treatment reduced Caspase-9 expression and was consistently associated with reduced cell death throughout the study, suggesting that bacterial supplements can counteract radiation-induced intestinal apoptosis.

Further, studies on mitigation strategies using BS have listed the positive impact on cancer patients receiving radiotherapy, including reduced diarrhoea-like symptoms and viral gastroenteritis^46^. Although probiotic treatment improved intestinal histology and reduced inflammatory damage, diarrhea persisted in some animals, suggesting that functional recovery of intestinal absorption and motility may lag structural repair. Radiation-induced alterations in electrolyte transport, water absorption, and microbial balance may contribute to the persistence of watery stools even after mucosal healing. Preclinical studies on bacterial supplementation with *Lactobacillus acidophilus*, *Lacticaseibacillus rhamnosus* GG ATCC 53103, and *Lactobacillus plantarum* 299v have shown improvements in small intestinal morphology, small intestinal crypt survival, and increased collagen content, respectively^7^. Additionally, the proposed mechanism of action of bacterial supplementation includes enhancing the intestinal immune system response, producing inhibitory agents, maintaining the intestinal integrity, maintaining a normal level of SCFAs, upregulating the electrolyte absorption of the intestine, modulating the function of the immune system, such as by suppressing the proinflammatory cytokines in the intestine, cell antagonism, and bacteriocin production^47,48^.

Our findings show that the formulated bacterial supplements effectively preserved the structural features of the gut, including jejunum villus height and crypt depth, and reduced radiation-induced fibrosis. Moreover, these supplements-maintained jejunum integrity at the cellular level and prevented molecular-level damage to the gut barrier by preserving TJ proteins. Further, PR-TR treatment reduced the expression of genes involved in the inflammatory response and apoptosis. Moreover, the current study had several limitations, including a relatively small sample size (n = 3) per group and a lack of in-depth mechanistic insights into the signalling pathways that modulate these gene expressions. While qRT-PCR provided transcriptional evidence, comprehensive protein-level validation (e.g., Western blot analysis of key markers such as NF-κB p65, phosphorylated p38 MAPK, cleaved caspase-3) was not performed, which excludes confirmation of post-transcriptional regulation and pathway activation. Future studies incorporating larger cohorts and comprehensive microbiome profiling will be essential to validate and expand upon these findings.

## Materials and methods

### Experimental design

Adult male Sprague Dawley (SD) rats (3–4 weeks old, 200–250 g) were housed and maintained at the Central Animal Facility (CPCSEA No: 94/PO/Re Bi/5/99/CPCSEA), Manipal Academy of Higher Education, Manipal, India. Animals were kept under controlled environmental conditions: temperature (23 ± 2 °C), humidity (50 ± 5%), and a 12-h light/dark cycle, with ad libitum access to standard feed and water.

All experimental procedures were conducted in accordance with ARRIVE guidelines and regulations, and followed the standards set by the World Health Organization (WHO), Switzerland, and the Indian National Science Academy (INSA), New Delhi. Ethical approval for the study was obtained from the Institutional Animal Ethics Committee, Manipal Academy of Higher Education (IAEC/KMC/84/2020).

Rats were divided into five groups, each consisting of six animals: the control group (C); the radiation group (R) underwent pelvic irradiation with 6 Gy on the 10^th^ day; the Prophylactic-Therapeutic (PR-TR) group was subjected to bacterial supplementation throughout the experiment as well as pelvic irradiation of 6 Gy on the 10^th^ day; and the Therapeutic (TR) group underwent pelvic irradiation on the 10^th^ day followed by bacterial supplementation (Fig. 1A). Following experimental duration, the animals were anaesthetised by intraperitoneal injection of Sodium Thiopentone at a dose of 40 mg/kg. Following anaesthesia, transcardial perfusion was performed with 0.9% saline, and the intestinal tissues were subsequently collected and stored for histopathological and other molecular assays.

### Pelvic irradiation procedure

Animals in the radiation and combination groups were kept in restrainers and irradiated with 6 Gy to the pelvic region on the 10^th^ day of the experimental schedule using an Elekta Medical Linear Accelerator (Versa H, Sweden) delivering 6 MV photon energy at Shirdi Saibaba Cancer Hospital, Manipal (Fig. 1B). Before irradiation, a dose delivery simulation was conducted on the restrainer using MONACO TPS 5.11 (Elekta, Sweden), employing an isocentric technique to ensure precise irradiation of the centre of interest within the 22 × 10 cm^2^ field. Radiation was delivered at 6 Gy/min through two beams: 3 Gy from the anterior–posterior (AP) direction and 3 Gy from the posterior-anterior (PA) direction. Setting the initial gantry angle to zero degrees for AP and 180 degrees for PA guaranteed accurate alignment of the radiation beam with the pelvic region. Furthermore, both the collimator and couch angles were set to 0° to maintain this alignment.

### Multi-strain bacterial combination

Based on our earlier study on protection against whole-body radiation exposure, the bacterial composition used for supplementation in the present study was selected^17^. The commercially available bacterial supplementation  (Fourrts India Laboratories Pvt. Ltd., India) contained the following bacterial species per dose: *Lactobacillus acidophilus* (2 × 10⁸ CFU), *Lacticaseibacillus rhamnosus* (1 × 10⁸ CFU), *Lacticaseibacillus casei* (1 × 10⁸ CFU), *Lactobacillus delbrueckii* subsp. *bulgaricus* (1 × 10⁸ CFU), *Lactiplantibacillus plantaru*m (1 × 10⁸ CFU), *Bifidobacterium longum* (1 × 10⁸ CFU), *Bifidobacterium breve* (1 × 10⁸ CFU), *Bifidobacterium infantis* (1 × 10⁸ CFU), and *Streptococcus thermophilus* (1 × 10⁸ CFU). The probiotic mixture was administered orally at a dose of 1 × 10⁹ CFU/kg body weight in a standardised volume of 1 ml /kg body weight. Animals were monitored daily for changes in body weight and faecal consistency.

### Histopathological analysis

The jejunum and colon samples were fixed for 48 h in a 10% neutral buffered formalin solution. Following fixation, specimens were embedded in paraffin blocks, and 5 µm sections were taken using a microtome (Leica RM2125RT, Wetzlar, Germany) for further staining.

#### Hematoxylin and eosin staining

Hematoxylin and eosin (H&E) staining was performed to evaluate the general tissue morphology and assess the structural alterations in the intestinal epithelium. In brief, the slides containing tissue sections underwent deparaffinization and rehydration, followed by hematoxylin staining for 1 min. Subsequently, they were counterstained with eosin for 15 s. Imaging was performed using a light microscope (Olympus CKX53, Tokyo, Japan) at 100X magnification. Morphological parameters, such as villus height, crypt depth, and mucosal thickness, were quantified using the ImageJ software for 10 intact, well-oriented villi and 10 crypts per Sect. (3–4 sections per animal) in the jejunum and colon region.

#### Periodic acid staining (PAS)

Periodic Acid–Schiff (PAS) staining was performed to evaluate goblet cell distribution and mucosal barrier integrity in intestinal tissues. The sections were subjected to oxidation in a 1% solution of periodic acid for 10 min and immersed in Schiff’s reagent for 10 min, following a counterstain treatment with hematoxylin for 1–2 min. Subsequently, images were captured using a light microscope at 200X magnification, and 10 well-oriented intact villi from 4 sections per animal (n = 3) were scored for goblet cells to analyse intestinal integrity.

#### Masson’s trichrome staining

Masson’s Trichrome staining was carried out to assess collagen deposition and fibrosis in intestinal tissues following irradiation. After deparaffinization and rehydration, the formalin-fixed tissue was refixed for 1 h in Bouin’s solution and maintained at 56 °C. After refixation, the tissue sections were stained with Weigert’s iron hematoxylin solution, followed by counterstaining with Biebrich scarlet–acid fuchsin solution. The differentiation process was carried out using a phosphomolybdic–phosphotungstic acid solution. Consequently, the sections underwent staining with an aniline blue solution. The quantitative measurement of staining intensity in specific target areas within 3–4 sections per animal at 100X magnification was carried out based on the grayscale threshold described earlier^49^.

#### Immunohistochemistry (IHC)

IHC was performed to analyse the expression of tight junction (TJ) proteins, Occludin and Zonula Occludens. The intestinal tissue sections were incubated with primary antibody at 4 °C (ZO-1; Novus; USA; 1:500, Occludin; Novus; USA; 1:500) and then incubated with 3% hydrogen peroxide. Furthermore, a secondary antibody, HRP-conjugated goat anti-rabbit IgG (Invitrogen, California, USA, 1:1000), was added. The sections were then incubated with 3,3′-diaminobenzidine (DAB) chromogen and counterstained with hematoxylin. From each animal, 3–4 sections (n = 3) were immunostained, and the optical density (OD) score of both TJ proteins was quantified^50^.

### Gene expression analysis

Total RNA from the jejunum was extracted by TRIzol (Thermo Fisher Scientific, USA) and reverse-transcribed to cDNA using a high-capacity cDNA Reverse Transcription kit (Applied Biosystems, USA). Optimum primers were designed using Primer3, and in silico PCR was performed to assess primer efficiency and estimate the expected product size using the UCSC Genome Browser (Supplementary Table 1). Quantitative RT‒PCR was performed for *Il-6, Ifn-γ, Bax, Bcl2,* and *Caspase-9* in Quant-Studio 6 pro (Applied Biosystems, USA) with SYBR green master mix (Qiagen, Germany) following the manufacturer’s protocol (Supplementary Table 2).

### Enzyme-linked immunosorbent assay (ELISA) for inflammatory proteins

Jejunum tissues from rats were minced in ice-cold PBS and homogenised at a ratio of 100 mg tissue to 1 mL PBS. The resulting homogenate was transferred to 1.5 mL Eppendorf tubes and centrifuged at 5000 g for 10 min at 4 °C. The supernatant was used for further analysis. Interleukin-6 (IL-6) and interferon-gamma (IFN-γ) levels were quantified using ELISA kits (Elabscience Biotechnology, USA) according to the manufacturer’s instructions.

### Statistical analysis

All analyses were performed using GraphPad Prism software (version 9; GraphPad Software, USA). The data were represented as the mean ± SEM, fold-change, and differences among groups were assessed using one-way analysis of variance (ANOVA). Statistical significance was determined at p < 0.05.

## Conclusion

The current investigation provides critical insights into the use of bacterial supplementation to mitigate radiation-induced changes in GI tract morphology, intestinal permeability, gastrointestinal fibrosis, inflammation, and apoptosis. While diverse strategies have emerged to mitigate radiation-induced GI toxicity, our study findings suggest that the formulated bacterial supplementation holds promise for addressing radiation-induced GI tract impairments in patients undergoing pelvic radiotherapy, thereby promoting host well-being. Microbiome-based therapies are sustainable, safe healthcare interventions because they use strains naturally present in the human gut. They have been extensively studied for their safety profile. Research has demonstrated their ability to exert beneficial effects on host health, including supporting gastrointestinal function and modulating the immune system. The results of our study strongly indicate that the administered bacterial supplementation may alleviate radiation-induced GI toxicity.

## Supplementary Information

Supplementary Information.

## Data Availability

The data can be requested from the corresponding author at [kamalesh.m@manipal.edu](mailto:kamalesh.m@manipal.edu).

## Abbreviations

GIT: Gastrointestinal tract (GIT)
BS: Bacterial supplementation (BS)
C: Control
R: Radiation
PR-TR: Prophylactic-therapeutic
TR: Therapeutic
Gy: Gray
PAS: Periodic acid staining
IHC: Immunohistochemistry
TJ: Tight junction
ZO-1: Zonula occludens-1
IL-6: Interleukin-6
INF-γ: Interferon gamma
Bax: Bcl2 associated X protein
Bcl2: B- cell leukemia/ lymphoma 2 protein
ELISA: Enzyme-linked immunosorbent assay
