# Supplementary material for: Multi-strain bacterial combination mitigates pelvic irradiation-induced gut damage by preserving gut integrity, inhibiting inflammation and apoptosis
Source: Sci Rep. 2026 Apr 15;16:17608. doi: 10.1038/s41598-026-47773-3 (PMC13243460; doi:10.1038/s41598-026-47773-3)
Supplement: Supplementary file 1 — Supplementary Information. [file 41598_2026_47773_MOESM1_ESM.docx]

**Supplementary Table 1. qRT-PCR primer sets**

| Gene | Sequence |
| --- | --- |
| *Il-6* | F (5’-GCAAGAGACTTCCAGCCAGT-3’)  R (5’-GTCTGTTGTGGGTGGTATCC-3’) |
| *Ifn-γ* | F (5’-GGCCTAGCTCTGAGACAATG-3’)  R (5’-GAGTGTGCCTTGGCAGTAAC-3’) |
| *Bax* | F (5’-GTTGCCCTCTTCTACTTTG-3’)  R (5’-AGCCACCCTGGTCTTG-3’) |
| *Bcl2* | F (5’-CGGGAGAACAGGGTATGA-3’)  R-(3’-CAGGCTGGAAGGAGAAGAT-3’) |
| *Caspase-9* | F (5’-GTAAACTTTGGCGGACTG-3’)  R (5’-AGCCTCTGAAATAGCACC-3’) |
| *Gapdh* | F (5’-CTTGCCTCTCAGACAATGCC-3’)  R (5’-ATACACACTCTGGGGCTGTC-3’) |

**Supplementary Table 2: qRT**-**PCR cycling conditions**

| **Stage** | **Temperature (°C)** | **Time** | **Cycles** |
| --- | --- | --- | --- |
| Initial hold | 50 | 2 min | 1 |
| Enzyme activation | 95 | 10 min | 1 |
| Denaturation | 95 | 15 s | 40 |
| Annealing/Extension | 60 | 1 min | 40 |
| Melt curve - Denaturation | 95 | 15 s | 1 |
| Melt curve - Annealing | 60 | 1 min | 1 |
| Melt curve - Dissociation | 95 | 1 s | 1 |

(Instrument: QuantStudio™ 6 Pro Real-Time PCR System (Applied Biosystems, USA), Chemistry: SYBR Green, Reaction volume: 10 µL).
